# Obesity increases eosinophil activity in asthmatic children and adolescents

**DOI:** 10.1186/1471-2466-13-39

**Published:** 2013-06-18

**Authors:** Milena Baptistella Grotta, Dalize M Squebola-Cola, Adyleia ADC Toro, Maria Angela GO Ribeiro, Silvia B Mazon, Jose D Ribeiro, Edson Antunes

**Affiliations:** 1 Paediatric, State University of Campinas (UNICAMP), Campinas, São Paulo, Brazil; 2 Pharmacology Department, State University of Campinas (UNICAMP), Campinas, São Paulo, Brazil

## Abstract

**Background:**

A clear relationship between asthma and obesity has been reported, but the mechanisms remain unclear. The aim of this study was to evaluate the influence of obesity on eosinophil activity (chemotaxis and adhesion) in asthmatic children and adolescents compared with cells from healthy volunteers.

**Methods:**

Asthmatic obese (AO), asthmatic non-obese (ANO), non-asthmatic obese (NAO) and non-asthmatic non-obese (NANO) individuals were included in the present study. The chemotaxis of eosinophils after stimulation with eotaxin (300 ng/ml), platelet-activating factor (10 μM; PAF) and RANTES (100 ng/ml) was performed using a microchemotaxis chamber. The eosinophil peroxidase activity was measured to determine the adhesion activity of eosinophils cultivated on fibronectin-coated plates. The serum leptin, adiponectin, TNF-α and IgE levels were quantified using ELISA assays.

**Results:**

The serum IgE levels and eosinophil counts were significantly higher in asthmatic (obese and non-obese) individuals compared with non-asthmatic individuals (obese and non-obese). Spontaneous eosinophil chemotaxis was greater in the AO group compared with either the ANO or NANO groups. The activation of eosinophils using eotaxin and PAF increased eosinophil chemotaxis in the AO group. RANTES treatment increased eosinophil chemotaxis in the NAO group compared with the NANO or ANO groups. The activation of eosinophils using eotaxin significantly increased eosinophil adhesion in the AO group compared with other groups. The serum leptin and TNF-α levels were higher in obese subjects (asthmatic and non-asthmatic), whereas the levels of adiponectin did not significantly differ among these groups.

**Conclusion:**

This study is the first to show increased eosinophilic activity (chemotaxis and adhesion) associated with high serum leptin and TNF-α levels in atopic asthmatic obese children and adolescents compared with non-obese healthy volunteers.

## Background

Recent meta-analyses [9,10], systematic reviews [11-13] and cross-sectional [1-5], case control [6] and prospective cohort [7,8] studies have demonstrated a relationship between asthma and obesity. High body mass index (BMI) has been associated with the increased incidence and prevalence of asthma, asthma severity, reduced responses to standard asthma medications, persistent symptoms and poorly controlled disease [14-16], suggesting a specific phenotype of asthma in obese individuals [17,18]. Obesity increases the risk of asthma in both sexes and in different ethnic groups. Several factors have been proposed, including obstruction of upper airways flows, gastroesophageal reflux, inconsistent breathing from sleep-disorders, the relationship between physical and sedentary activity, genetics and the state of low-grade systemic inflammation through obesity [19,20]. However, the exact mechanisms responsible for the relationship between obesity and asthma remain unknown.

Eosinophils are the primary effector cells responsible for ongoing airway inflammation in atopic asthmatic individuals [21]. Evidence suggests that the recruitment of eosinophils to sites of inflammation is a multifactorial and multistep process, involving eosinophil-endothelial interactions through adhesion molecules and the local generation of chemotactic agents that direct cell migration into the inflamed airways. Previous studies have suggested that the cytokines IL-3 and IL-5, granulocyte/macrophage-colony stimulating factor (GM-CSF) and adipokines are involved in this process [22]. Eosinophils migrate along the concentration gradient of chemoattractants, enter pulmonary circulation, marginate the vessel wall and subsequently enter the interstitial spaces. However, the mechanisms by which obesity enhances the clinical expression of asthma-related physiological changes have not been fully elucidated. In murine models of diet-induced obesity and allergic diseases, ovalbumin challenge promotes hyperresponsiveness and eosinophilic inflammation associated with increased lung Th2 cytokines, serum IgE and lung parenchyma remodelling [23-25]. However, to the best of our knowledge, there are no studies concerning in vitro eosinophilic activities (chemotaxis and adhesion) in asthmatic obese individuals. Recent studies reported that the number of eosinophils in sputum or serum does not significantly differ between obese or non-obese asthmatics [26,41]. Thus, we hypothesised that the increase in obesity-associated systemic inflammatory mediators activates eosinophils, thereby exacerbating pulmonary inflammation, which is a direct component of asthma pathophysiology. Therefore, the aim of this study was to evaluate the influence of obesity on peripheral blood eosinophil functions (chemotaxis and adhesion) in asthmatic children and adolescents.

## Methods

### Subjects

A cross-sectional study was performed with 6-18-year-old outpatients from the Paediatric Asthma Clinic of State University of Campinas (UNICAMP). This study was approved through the Ethics Committee of State University of Campinas (UNICAMP). – (Agreement 352/2010). All patients and parents or guardians provided informed consent before beginning the study.

We selected four groups of individuals, namely asthmatic obese (AO, n = 16), asthmatic non-obese (ANO, n = 16), non-asthmatic obese (NAO, n = 5) and non-asthmatic non-obese (NANO, n = 5) individuals. The asthma diagnosis was established according to the criteria of the American Thoracic Society-European Respiratory Society (ATS-ERS) [27], and the degree of asthma was classified according to the Global Initiative for Asthma (GINA) as intermittent, mild persistent, moderate persistent and severe persistent asthma [28]. All patients were atopic and sensitised to common perennial inhaled allergens, as evaluated through skin-prick tests. The patients received regular follow-up examinations at the paediatric pulmonology outpatient clinic, with asthma treated using inhaled corticosteroids (ICS), according to GINA [28] that is 400 mcg/day for mild asthma, 400 mcg ICS plus 12 mcg long-acting beta agonist (LABA) for moderate asthma and 800 mcg/day ICS plus 24 mcg/day LABA for severe asthma. The patients were not smokers.

Obesity was defined as a body mass index (weight (kg)/ height (m^2^)) above 95 percentile, according to the NCHS (National Centre for Health Statistics) BMI curve [29]. The control group (NANO) comprised healthy volunteers with normal lung function and without diagnostic criteria for asthma and obesity. The non-asthmatic obese group (NAO) did not present diagnostic criteria for asthma, but exhibited BMIs above the 95th percentile. In healthy children/adolescents, due to the smaller number of blood eosinophils, a higher volume of blood (60 ml) was required to perform the functional assays in vitro; therefore, for ethical reasons, 5 individuals were included in these groups. The exclusion criteria included children younger than 6 years old due to their inability to perform the lung function test and the presence of co-morbidities, respiratory infections or uncontrolled asthma during the previous 4 weeks. All patients were treated with anthelmintic albendazole at 400 mg (10 ml) in a single dose for one month before beginning the study, excluding eosinophilia due to parasitosis. The serum cholesterol, triglycerides, fasting glucose and IgE levels and circulating eosinophil counts were obtained from each patient.

### Eosinophil isolation

The eosinophils were isolated from peripheral blood as previously described [30]. Briefly, 30 ml of blood, collected in 3.13% (w/v) sodium citrate, was diluted 1:1 using phosphate buffered saline (PBS), and 35 ml of diluted blood was overlaid onto a 15-ml Percoll gradient (Sigma Chemical Co-USA). The gradients were centrifuged at 1000 *g* for 23 min at 4°C (Hermle model Z360k centrifuge, Germany), and the pellet containing red cells and granulocytes were collected. The red cells were lysed using lysing buffer, and the remaining granulocytes were washed. Another lysis was performed using distilled water. Subsequent to centrifugation at 400 *g* for 10 min at 4°C, and subsequently the pellet was incubated with anti-CD16 immunomagnetic microbeads for 30 min before passing on a steel-matrix column in a magnetic field, and the CD16-negative eosinophils were collected. The eosinophils were resuspended in minimum essential medium (MEM), pH 7.2 (> 92% eosinophils; the contaminating cells were mononuclear cells).

### Chemotaxis assays

The eosinophils were resuspended at a concentration of 4 × 10^6^ cells/ml in minimum essential medium (MEM), and migration assays were performed using a 48-well microchemotaxis chamber [31]. The bottom wells of the chamber were filled with the chemoattractants eotaxin (300 ng/ml), PAF (10 μM) and RANTES (100 ng/ml) or MEM (control), and the upper wells were filled with eosinophils (50 μl). The bottom and upper cells were separated using a 5-μm polycarbonate filter (Nucleopore, Pleasanton, CA, USA). The chamber was subsequently incubated for 1 h at 37°C (5% CO_2_). At the end of the incubation period, the filter was removed, washed, stained with Diff- Quik® (Baxter Healthcare Corporation, USA), and mounted on a glass slide. The incubations were performed in triplicate, and migration was determined by counting the eosinophils that migrated completely through the filter in five random high-powered fields (HPF).

### Eosinophil adhesion assay

The adhesion assay was performed as previously described [32]. Briefly, 96-well plates (Maxisorp, Nunc, Roskilde, Denmark) were prepared by coating individual wells with 60 μl of fibronectin overnight at 4°C. The wells were washed twice with PBS before blocking the non-coated sites using 0.1% (w/v) bovine serum albumin (BSA) for 60 min at 37°C. The wells were washed twice with PBS, and the plates were dried. The eosinophil suspension was subsequently incubated for 30 min at 37°C (5% CO_2_) with the adhesion stimulus eotaxin (100 ng/ml). The treated eosinophils were added at a volume of 50 μl of MEM (7 × 10^4^ cells/ml) to the coated wells of a 96-well plate. The cells were incubated to adhere to the wells for 30 min at 37°C (5% CO_2_). After incubation, the non-adhered cells were removed, and the remaining cells were washed twice with PBS. Fifty microliters of MEM was added to each well and varying concentrations of the original cell suspension (in MEM) were added to the empty wells to form a standard curve. The eosinophil adhesion was calculated after measuring the residual eosinophil peroxidase (EPO) activity of adherent cells. Briefly, EPO substrate (Sigma Chemical Co., St Louis, MO) was added to each well and incubated for 30 min at room temperature. The reaction was terminated using 25 μL H_2_SO_4_ (4 M) per well, and the absorbance was measured at 490 nm in a microplate reader (Multiscan MS; Labsystems, Helsinki, Finland). The adherence was calculated by comparing the absorbance of the unknown samples to that of a standard curve.

### Serum leptin, adiponectin and TNF-α levels

After centrifugation, 2 ml aliquots of serum were frozen (−60°C). The leptin, adiponectin and TNF-α levels were measured in serum using commercially available enzyme-linked immunosorbent assay (ELISA) kits according to the manufacturer’s instructions (Millipore, St Charles, Missouri, USA). The leptin and adiponectin levels ranged from 0.5 to 100 and 1.56 to 100 ng/mL, respectively. Low and high quality controls were assayed in parallel in each assay.

### Measurement of lipid levels, fasting glucose and IgE

The levels of total cholesterol, high-density lipoprotein (HDL) and triglycerides in serum were measured using commercially available kits (Roche Laboratories, São Paulo, Brazil). The low-density lipoprotein (LDL) levels were calculated according to the manufacturer’s instructions. The blood glucose concentration was measured (Accu-Check Performa, Roche Diagnostics, Indianapolis, IN, USA). The IgE kit was obtained from Dade Behring (Marburg, USA).

### Statistical analysis

The sample size was derived from a pilot project including a small group of obese and non-obese subjects. The study was designed to have 80% power to detect a significant difference between obese and non-obese individuals, with a type I error of 5%. The data are presented as the mean values ± SEM of *n* experiments. The Instat (GraphPad software) programme and the SAS System for Windows (Statistical Analysis System, version 9.2, SAS Institute, Inc, 2002–2008, Cary, NC, USA) were used for the statistical analysis. One-way ANOVA followed by Turkey`s test was used to analyse cholesterol, triglycerides, fasting glucose, serum IgE, eosinophil counts, chemotaxis, adhesion, and the leptin and adiponectin levels. The adiponectin levels were analysed using ANOVA, followed by the Kruskal-Wallis test. P < 0.05 was accepted as statistically significant.

## Results

### Subject characteristics and serum measurements

The characteristics of the study subjects are presented in Table 1. There were no differences in the age or sex among the groups. The total cholesterol was elevated in the obese groups (asthmatic and non-asthmatic) compared with non-obese individuals (asthmatic and non-asthmatic; P = 0.0009). The levels of HDL, VLDL and LDL-cholesterol, triglycerides and fasting glucose were not significantly different between groups.

**Table 1:** Subject characteristics

	**Asthmatic obese (AO)**	**Non-asthmatic obese (NAO)**	**Asthmatic non-obese (ANO)**	**Non-asthmatic non-obese (NANO)**	**P value**
N	16	5	16	5	
Age (years)	10	11	9.5	28	0.6804
Sex (% males)	56%	40%	62.5%	40%	0.7832
Total cholesterol	176	214.2(a)	136.75(a)	111.8	0.0009
BMI	28.16 ± 4.46	31.54 ± 4.12	18.79 ± 3.39	21.55 ± 1.63	0.0050
FVC	102.5	102.8	94	90	0.3172
FEV_1_	91	102	86	98	0.2581
FEV_1_/FVC	88	92.6	87.5	93	0.0752
HDL	42.5	40.2	46.94	48	0.2919
VLDL	15.5	24	17	14.4	0.2356
LDL	82.0	146.6(b)	90.38(b)	71	0.0360
Tryglicerides	77.0	120.8	76.88	83.6	0.1617
Glucose (g/dL)	81.0	79	79.81	81.6	0.7901
IgE (U/mL)	653.5(c)	84.8	862.67(c)	89.8	0.0007
Blood eosinophil (%)	7.05(d)	5.06	8.31(d)	1.52	0.0024

Data presented as median or percentage (%) of subjects with the specified factor.

(a) and (b) p < 0.05 – versus to non-obese groups.

(c) and (d) p < 0.05 – versus to non-asthmatic groups.

The serum IgE levels and eosinophil counts were significantly higher in the asthmatic individuals (obese and non-obese) compared with the non-asthmatic individuals (obese and non-obese; P = 0.0007 for IgE; P = 0.024 for eosinophil counts) (Table 1).

### Eosinophil chemotaxis to eotaxin, PAF and RANTES

**Figure 1:**
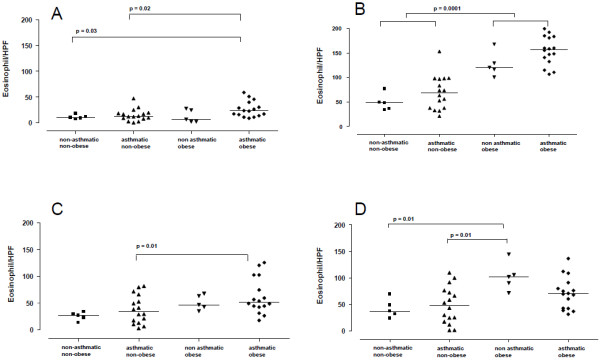
**Human eosinophil chemotaxis in asthmatic obese (AO), asthmatic non-obese (ANO), non-asthmatic obese (NAO) and non-asthmatic non-obese (NANO) subjects in response to (A) minimum essential medium (MEM, 27 μL), (B) eotaxin (300 ng/ml), (C) PAF (10 μM) and (D) RANTES (100 ng/ml).** Eosinophils (4 × 10^6^/ml /50 μl / well) were incubated for 1 h at 37°C in the microchemotaxis chamber, and the filters were stained and mounted on a glass slide. Eosinophil migration was determined after counting the eosinophils that migrated completely through the filter in five random high-powered fields (HPF; 1000 x) per well. The results are expressed as the mean migrated cells for each individual (P values are shown in figure).

Figure 1A shows that the spontaneous eosinophil chemotaxis (non-activated cells; MEM) was increased in the asthmatic obese (AO) group compared with either asthmatic or non-asthmatic non-obese individuals (NAO and NANO groups, respectively).

The activation of eosinophils using eotaxin (300 ng/ml; Figure 1B) and PAF (10 μM; Figure 1C) also increased cell chemotaxis in the asthmatic obese (AO) group compared with ANO and NANO groups. The increased eotaxin- and PAF-induced chemotaxis in AO individuals did not significantly differ from that observed in the NAO groups (Figure 1B,C). With regard to RANTES (100 ng/ml; Figure 1D), increased cell chemotaxis was observed in the non-asthmatic obese (NAO) group compared with either non-asthmatic (NANO group) or asthmatic (ANO group) non-obese individuals. No significant differences were detected between the NAO and AO groups (Figure 1D).

### Eosinophil adhesion to fibronectin-coated plates

**Figure 2:**
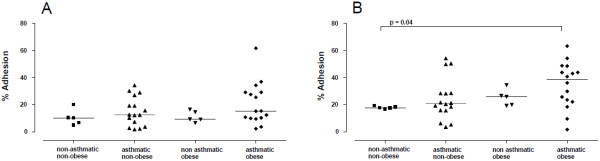
**Adhesion of human eosinophils to fibronectin coated plates.** Eosinophils (7 × 10^4^ cells/ml) of asthmatic obese (AO), asthmatics non-obese (ANO), non-asthmatic obese (NAO) groups and healthy volunteers (NANO) were incubated with MEM (**A**) and eotaxin (**B**), and subsequently incubated to evaluate the adherence to fibronectin-coated wells for 30 min. The results are expressed as the mean adhered cell percentages for each individual (P values are shown in figure).

Basal eosinophil adhesion to human fibronectin was similar among all groups (Figure 2A). The activation of eosinophils with eotaxin (100 ng/ml) significantly increased the adhesion of eosinophils in the AO group compared with NANO, ANO and NAO subjects (Figure 2B).

### Levels of leptin, adiponectin and TNF-α

**Figure 3:**
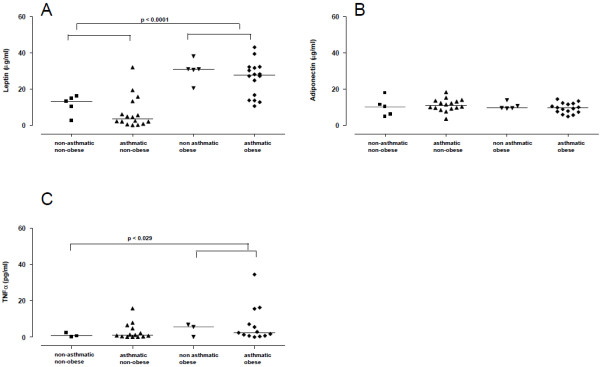
**Leptin (A), adiponectin (B) and TNF-α (C) levels in the serum of asthmatic obese (AO), asthmatics non-obese (ANO), non-asthmatic obese (NAO) groups and healthy volunteers (NANO).** Adipokines and TNF-α levels were measured in the serum using the commercially available enzyme-linked immunosorbent assay (ELISA) kits (P values are shown in figure).

Figure 3A shows that serum leptin levels were higher in obese subjects (asthmatic and non-asthmatic) compared with non-obese groups (NANO and ANO; P < 0.0001). Serum adiponectin levels did not significantly differ among the groups (Figure 3B). The serum TNF-α levels were higher in obese subjects (asthmatic and non-asthmatic) compared with non-asthmatic non-obese subjects (P < 0.029; Figure 3C).

## Discussion

The present study is the first to show increased eosinophilic activity (chemotaxis and adhesion) in atopic obese individuals associated with high serum leptin and TNF-α levels in children and adolescents compared with non-obese volunteers. The patients received regular follow-up examinations in paediatric pulmonology outpatient clinics, with asthma control and the regular use of inhaled corticosteroids, according to severity classifications. Despite the clinical control and lung function test, circulating eosinophils in asthmatic obese individuals (AO group) exhibited an increased level of pre-activation, as evidenced through increased chemotaxis and adhesion in these individuals.

Eotaxin is the strongest and the most specific factor for eosinophils, acting through interactions with CCR3 receptors [33]. The eosinophil counts in an infiltrated organ are proportional to the eotaxin concentrations at the inflammatory site [34-37], with poor asthma control and increased symptom severity. In addition, we also used the chemoattractants PAF and RANTES to evaluate the eosinophil activity in all groups of individuals. PAF is a well-known phospholipid mediator that regulates a variety of physiopathological processes in different cell types that might be important in the pathogenesis of asthma [38]. RANTES, a product of activated T cells, is elevated in the atopic and non-atopic asthmatic airways and promotes eosinophil and T cell infiltration. The expression of RANTES has been demonstrated in bronchial smooth muscle, eosinophils and T cells [39]. In the present study, spontaneous chemotaxis (non-activated cells) was elevated in asthmatic obese individuals compared with asthmatic non-obese individuals. Similarly, eotaxin- and PAF-induced eosinophil chemotaxis was increased in asthmatic obese individuals compared with asthmatic non-obese individuals. Interestingly, obesity itself (in the absence of asthma) increased eosinophil chemotaxis towards eotaxin and RANTES.

Adhesion molecules and chemokines play key roles in selective eosinophil accumulation. The integrins macrophage adhesion molecule-1 (Mac-1, CD11b/CD18, αMβ2) and very late antigen-4 (VLA-4, CD49d/CD29, α4β1) are important for the firm adhesion of eosinophils to the endothelium through the intercellular adhesion molecule (ICAM)-1 and the vascular cell adhesion molecule (VCAM)-1, respectively [39]. Both Mac-1 and VLA-4 integrins expressed in eosinophils bind to extracellular matrix components, such as fibronectin [39]. The firm adhesion of eosinophils to fibronectin is mediated through the binding of eosinophil-expressed VLA-4 to the connecting segment 1 (CS-1) region of fibronectin. The adhesion assays performed on fibronectin-coated plates revealed increased eotaxin-induced eosinophil adhesion in the asthmatic obese group compared with the other groups.

Circulating leptin levels were directly correlated with adipose tissue mass [40,41]. Leptin is also a pro-inflammatory mediator that enhances systemic and pulmonary inflammation [42]. Therefore, high levels of leptin are essential in linking obesity to allergic airway inflammation [43]. In human eosinophils obtained from healthy volunteers, leptin up-regulated the cell surface expression of the adhesion molecule ICAM-1, stimulated the eosinophil chemokinesis and induced the release of inflammatory cytokines [22]. Moreover, leptin up-regulates TNF-α production and adhesion molecule expression in endothelial cells [22]. Leptin exerts both direct and indirect effects on eosinophil chemotaxis and intracellular signalling. In physiological settings, leptin might maintain eosinophil accumulation at inflammatory foci [44]. In the present study, the serum leptin and TNF-α levels were higher in obese individuals, irrespective of the presence of asthma, suggesting that leptin might be involved in the priming of circulating eosinophils. The results of recent studies are inconclusive regarding the independent association between serum leptin concentrations and the risk for asthma. Studies performed in children had significantly smaller sample sizes, showing a positive association between these two factors [42], while other studies showed no association [45]. The adiponectin levels diminished with increased adiposity and during the inflammatory response [40,43]. The anti-inflammatory activities of adiponectin has been associated with the reduced production/activity of TNF-α and the inhibition of IL-6 accompanied by the induction of the anti-inflammatory cytokines IL-10 and IL-1 receptor antagonist [40]. However, in the present study, we did not observe statistically significant differences among the groups, ruling out the possibility that adiponectin regulate the in vitro activity of eosinophils.

A limitation of our study is that the healthy volunteers comprising the control group (NANO) are older (28 years) compared with AO (10 years), NAO (11 years) and ANO (9.5 years) groups. Therefore, we cannot rule out the possibility that the observed differences are due to an age effect.

## Conclusion

In conclusion, obesity associated with increased serum leptin and TNF-α levels enhance eosinophil chemotaxis and adhesion in asthmatic children and adolescents. Despite effective asthma control with the regular use of inhaled corticosteroids, there is an increased activity in circulating eosinophils from obese asthmatics. Thus, there is a pressing need to improve our understanding of the mechanisms underlying the relationship between obesity and asthma and to develop treatment strategies to improve the health outcomes of obese patients with asthma.

## Competing interests

The authors declare that they have no competing interests.

## Authors’ contributions

MBG: made substantial contributions to the study conception and design, data acquisition, and data analysis and interpretation and participated in drafting and revising the manuscript. JDR: participated in the design of the study and in the collection of clinical markers. DSC: performed the eosinophil chemotaxis and adhesion studies and drafted the manuscript. AADCT: participated in the design of the study and in the collection of clinical markers. MAGOR: participated in the design of the study and performed the spirometry analysis. SBM: analysed the serum leptin and adiponectin levels and participated in the study design. EA: participated in the study design, data analysis and interpretation and gave the final approval for the publishing of this version of the manuscript. All authors have read and approved the final manuscript.

## Pre-publication history

The pre-publication history for this paper can be accessed here:

http://www.biomedcentral.com/1471-2466/13/39/prepub
